# Bioinformatic analysis highlights SNHG6 as a putative prognostic biomarker for kidney renal papillary cell carcinoma

**DOI:** 10.1186/s12894-023-01218-5

**Published:** 2023-04-01

**Authors:** Yifu Liu, Xiaofeng Cheng, Ping Xi, Zhicheng Zhang, Ting Sun, Binbin Gong

**Affiliations:** 1 Department of Urology, The First Affiliated Hospital of Nanchang University, Nanchang, 330006 Jiangxi China; 2 Jiangxi Institute of Urology, Nanchang, 330006 Jiangxi China

**Keywords:** Small nucleolar RNA host gene 6, Kidney renal papillary cell carcinoma, Prognosis, Biomarkers

## Abstract

**Purpose:**

Kidney renal papillary cell carcinoma (KIRP) is a highly heterogeneous malignancy and current systemic therapeutic strategies are difficult to achieve a satisfactory outcome for advanced disease. Meanwhile, there is a lack of effective biomarkers to predict the prognosis of KIRP.

**Methods:**

Using TCGA, GTEx, UALCAN, TIMER, TIMER 2.0 and STRING databases, we analyzed the relationship of SNHG6 with KIRP subtypes, tumor-infiltrating immune cells and potential target mRNAs. Based on TCGA data, ROC curves, Kaplan–Meier survival analysis and COX regression analysis were performed to evaluate the diagnostic and prognostic value of SNHG6 in KIRP. Nomogram was used to predict 3- and 5-year disease-specific survival in KIRP patients. In addition, with the help of Genetic ontology and Gene set enrichment analysis, the biological processes and signalling pathways that SNHG6 may be involved in KIRP were initially explored.

**Results:**

In patients with KIRP, SNHG6 was significantly upregulated and associated with a more aggressive subtype (lymph node involvement, pathological stage IV, CIMP phenotype) and poor prognosis. The ROC curve showed good diagnostic efficacy (AUC value: 0.828) and the C-index of the Nomogram for predicting DSS at 3 and 5 years was 0.920 (0.898–0.941). In the immune microenvironment of KIRP, SNHG6 expression levels were negatively correlated with macrophage abundance and positively correlated with cancer-associated fibroblasts. Furthermore, SNHG6 may promote KIRP progression by regulating the expression of molecules such as AURKB, NDC80, UBE2C, NUF2, PTTG1, CENPH, SPC25, CDCA3, CENPM, BIRC5, TROAP, EZH2. Last, GSEA suggests that SNHG6 may be involved in the regulation of the PPAR signalling pathway and the SLIT/ROBO signalling pathway.

**Conclusions:**

Our analysis suggests that a high SNHG6 expression status in KIRP is associated with a poorer prognosis for patients, and also elucidates some potential mechanisms contributing to this poorer outcome. This may provide new insights into the treatment and management of KIRP in the foreseeable future.

## Introduction

Kidney renal papillary cell carcinoma (KIRP), is the most common type of non-clear cell renal cell carcinoma (non-ccRCC) in the worldwide, accounting for approximately 15–20% of all renal cell carcinomas [1]. The KIRP is histologically divided into two major types, with type I KIRP being more associated with MET mutations, while type II is considered to be more heterogeneous and subdivided into various subtypes based on the genetic and molecular composition of the tumor [2, 3]. At present, systemic treatment strategies for advanced renal cell carcinoma revolve around ccRCC, either with immune checkpoint inhibitors or targeted therapies. However, the relative lack of specific gene mutations in ccRCC, such as VHL and PBRM1, decreases response rates to targeted therapeutic strategies in KIRP patients [4, 5]. Meanwhile, immune checkpoint inhibitors have shown limited activity in the KIRP population [6]. Moreover, the heterogeneity of the tumor itself presents another major challenge for therapeutic strategies. In this context, it is essential to refine the molecular and immunological landscape of KIRP.

Long non-coding RNAs (LncRNAs) are an RNA transcription with over 200 nucleotides and no apparent protein coding capability [7]. Its overexpression, defects or mutations have been found to be associated with a variety of human diseases, including cancer, neurological disorders, cardiovascular diseases and more [8]. Small nucleolar RNA host gene 6 (SNHG6), a lncRNA located in chromosome 8q13.1, has been shown to be linked to poor outcomes in a variety of human cancers [9]. For instance, SNHG6 can promote the progression of colorectal and ovarian clear cell carcinomas by regulating the expression of enhancer of zeste homolog 2 (EZH2) through a ceRNA network [10, 11]. In xenograft mice with lung cancer, SNHG6 can also regulate the differentiation of myeloid-derived suppressor cells by inhibiting protein expression of EZH2 via ubiquitin [12]. Furthermore, SNHG6 can interact with heterogeneous nuclear ribonucleoprotein L (HNRNPL) and polypyrimidine tract binding protein 1 (PTBP1) to promote the progression of hepatocellular carcinoma [13]. Taken together, SNHG6 may be implicated in the regulation of tumor development by multiple pathways and is considered a poor prognostic biomarker for human cancers. Whereas, it is still unclear whether SNHG6 has a place in the tumor microenvironment of KIRP as well.

Here, based on the TCGA database, we were the first to analyze the expression levels of SNHG6 in KIRP, the association of SNHG6 with KIRP subtypes and its predictive value for KIRP. Subsequently, in the context of these assumptions holding true, we further explored the relationship between SNHG6 and the abundance of tumor-infiltrating immune cells in the tumor microenvironment, potential mRNAs downstream of SNHG6, and the biological processes and signalling pathways that may be involved in SNHG6 in KIRP.

## Methods

### Data access

Sequencing data and corresponding clinicopathological information for 288 KIRP samples and 32 adjacent normal samples were downloaded from the TCGA database (https://portal.gdc.cancer.gov/) (14). 136 SNHG6 expression data in normal kidney tissue samples were obtained from the GTEx database (https://www.gtexportal.org/) to compensate for the relative lack of normal samples when performing differential gene expression analysis [15].

### UALCAN database

UALCAN (http://ualcan.path.uab.edu/) is a comprehensive, publicly available web resource that encompasses multiple cancer OMICS data (including TCGA, MET500, CPTAC and CBTTC) [16]. We used it to comprehensively analyze the relationship between SNHG6 expression and age, gender, race, pathological stage and histological subtype of KIRP patients.

### Nomogram construction and evaluation

First, 282 patients with KIRP were included in univariate and multivariate COX regression analyses to evaluate the impact of clinicopathological information (age, gender, BMI, race, smoking, pathological stage, and SNHG6 expression levels) on patients' disease-specific survival (DSS). Subsequently, indicators that were statistically significant in the multivariate COX analysis were included in the construction of the Nomogram to predict patients' DSS at 3 and 5 years. The calibration curve drawn by the "rms" R package is used to evaluate the agreement between the predicted and actual values.

### TIMER databases

TIMER is a comprehensive resource containing information on the immune infiltration of multiple cancer types and is currently available in TIMER (https://cistrome.shinyapps.io/timer/) and TIMER 2.0 versions (http://timer.cistrome.org/)[17, 18]. We used these tools to explore the correlation of SNHG6 with tumor-infiltrating immune cells in KIRP; for statistically significant cell types, Kaplan–Meier curves were further plotted to analyze the impact of these correlations on overall survival.

### Target mRNA screening and protein–protein interaction (PPI)

On the one hand, we performed gene correlation analysis for SNHG6, and mRNAs with spearman's correlation coefficient > 0.3 were selected. On the other hand, we screened the differentially expressed genes (DEGs) in KIRP based on logFC > 1.5 and plotted the volcano plot. subsequently, the two sets of screened genes were analyzed by intersection analysis via a Venn diagram and the target molecules were used in the next step of PPI and genetic ontology (GO) analysis. PPI networks for the target mRNAs were constructed in the STRING database (https://cn.string-db.org/) and next imported into Cytoscape (version 3.9.1) to seek out more critical molecules.

### GO and gene set enrichment analysis (GSEA)

GO analysis was used to evaluate the biological processes, molecular functions, and cellular composition of the target mRNAs. GSEA explored the potential signalling pathways of SNHG6 in KIRP by gathering differential genes between the high and low expression of SNHG6 groups. 1000 operations were performed per analysis, with normalized enrichment score > 1, false discovery rate < 0.25 and a nominal P value < 0.05 being considered statistically significant. These were performed using the 'clusterprofiler' of the R package.

### Statistical analyses

RNAseq data obtained from TCGA and GTEx databases were analyzed using the R package (version 3.6.3). Wilcoxon signed rank test, Wilcoxon rank sum test and Kruskal–Wallis test were used for intergroup comparison and analysis of the relationship between SNHG6 and clinicopathological features. ROC curves and Kaplan–Meier analysis were used to assess the diagnostic and prognostic predictive value of SNHG6 for KIRP. Spearman correlation analysis was used to evaluate the correlation between SNHG6 expression levels with tumor-infiltrating immune cells and mRNA. p < 0.05 was considered statistically significant.

## Results

### Pan-cancer analysis of SNHG6

**Fig. 1:**
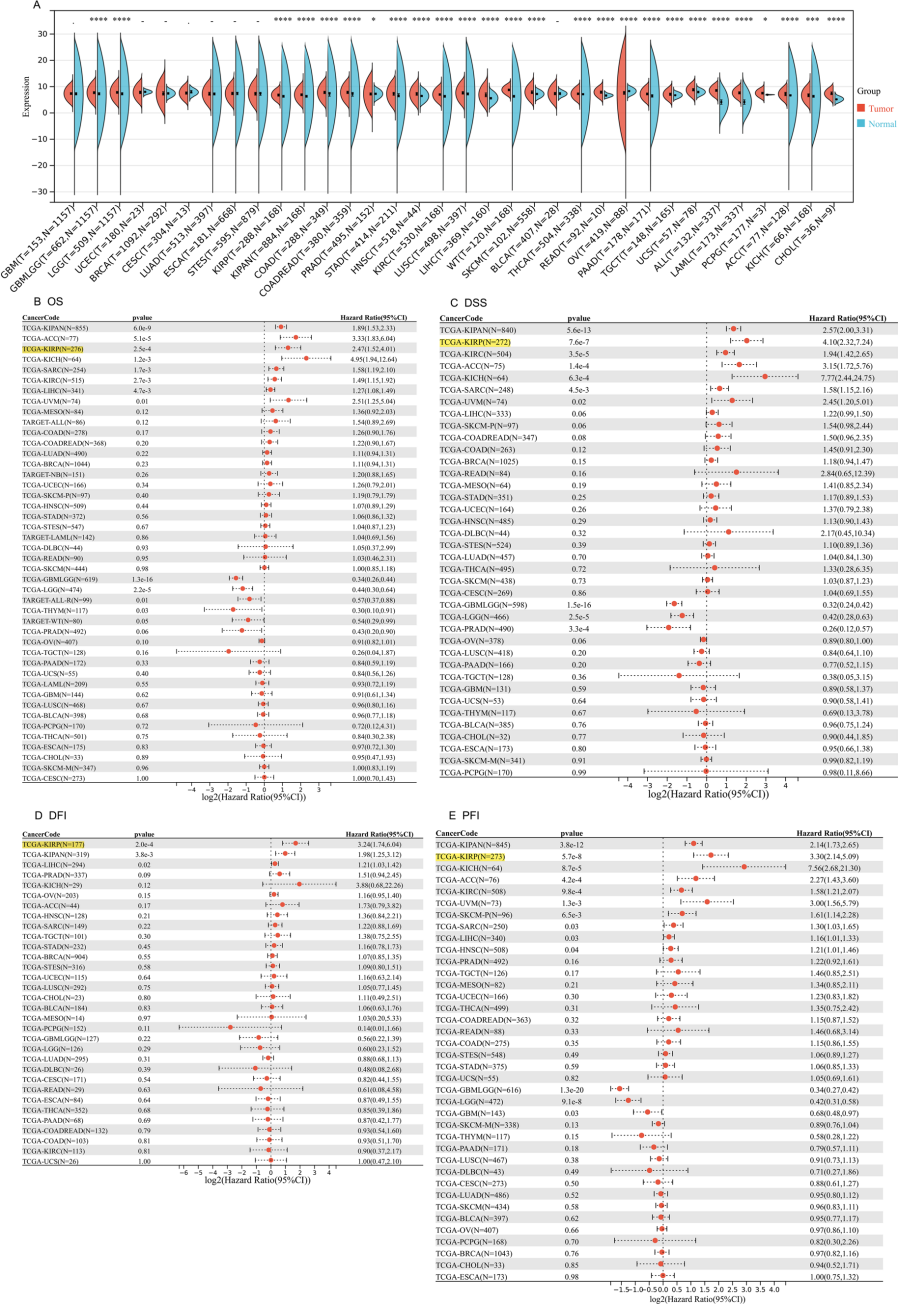
Expression of SNHG6 in pan-cancer and the effect of SNHG6 upregulation on the prognosis of human cancers. **A** SNHG6 expression is upregulated in a variety of cancer tissues. Effect of upregulation of SNHG6 expression on **B** OS, **C** DSS, **D** DFI and **E** PFI in human cancer patients. *, p < 0.05; **, p < 0.01; ***, p < 0.001; ****, p < 0.0001. SNHG6, Small nucleolar RNA host gene 6; KIRP, kidney renal papillary cell carcinoma; OS, overall survival; DSS, Disease-specific survival; DFI, disease-free interval; PFI, progression-free interval

By integrating genomic data from the TCGA database and GTEx database, we found that SNHG6 was highly expressed in a variety of human cancers, including glioma, renal cancer, gastric cancer, colorectal cancer, head and neck squamous cell carcinoma, lung squamous carcinoma and melanoma, etc. (see Fig. 1A for details). Subsequently, we comprehensively analyzed the effects of SNHG6 on overall survival (OS), DSS, disease-free interval (DFI), and progression-free interval (PFI) of cancer patients, and the results showed that upregulation of SNHG6 may be detrimental to the prognosis of KIRP patients. (Fig. 1B–E). Therefore, KIRP was chosen as the target cancer for this study.

### Expression levels of SNHG6 in KIRP and correlation with KIRP subtypes

**Fig. 2:**
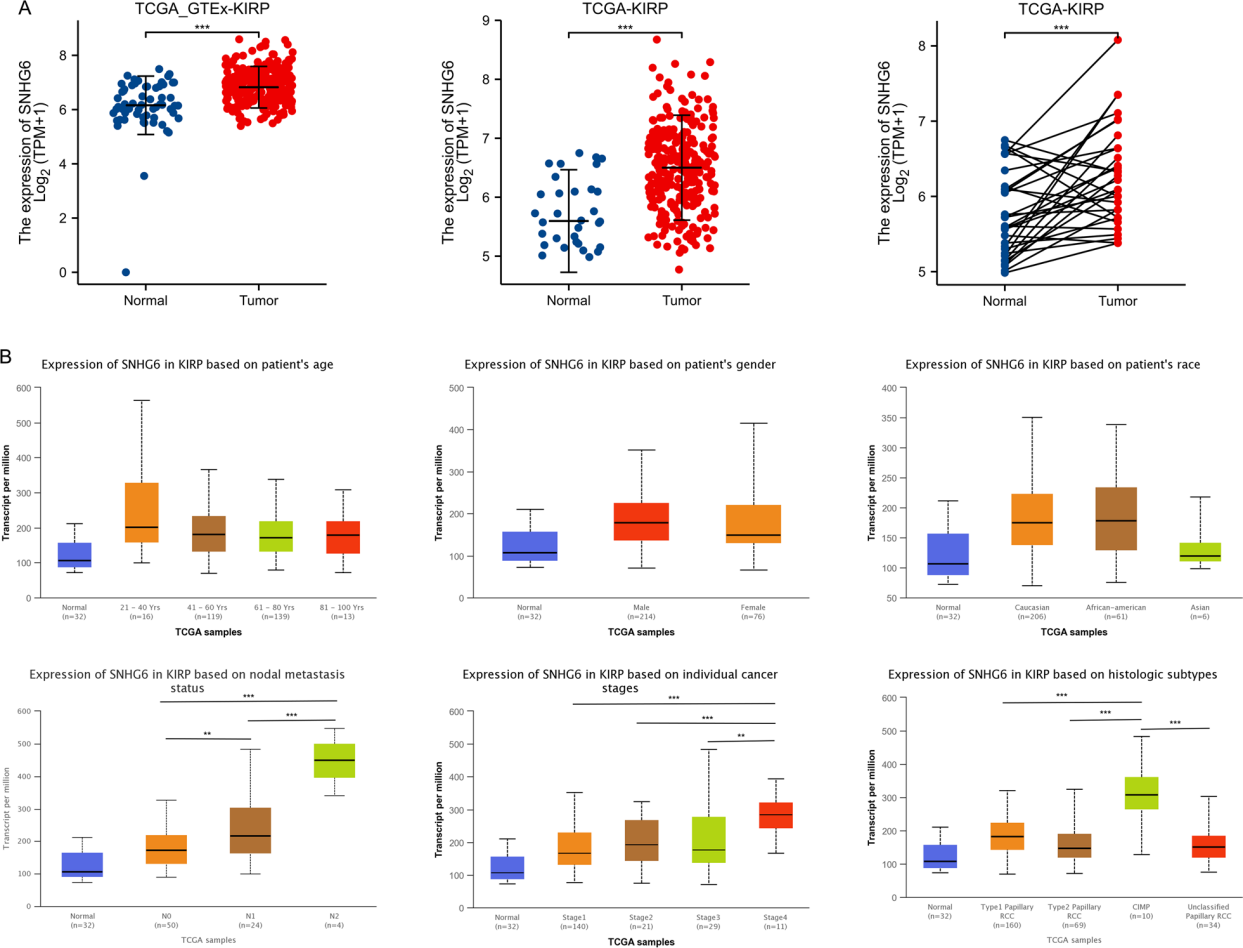
Expression of SNHG6 in KIRP and the relationship between SNHG6 expression and KIRP subtypes. **A** SNHG6 is highly expressed in KIRP tissues (based on TCGA and GTEx databases). **B** High SNHG6 expression was observed in patients with lymph node involvement, pathological stage IV, CIMP phenotype; and there was no significant correlation with gender, age and race. **, p < 0.01; ***, p < 0.001; ns, not significant. SNHG6, Small nucleolar RNA host gene 6; KIRP, kidney renal papillary cell carcinoma

As shown in Fig. 2A, SNHG6 was upregulated in KIRP tissues with or without the addition of data from the GTEx database. In the meantime, this phenomenon could be similarly observed in the 32 paired samples. Supported by this evidence, we further compared the expression levels of SNHG6 in different subgroups of KIRP. The results showed that the expression of SNHG6 was not significantly correlated with the age, gender and racial differences of the patients, while it was significantly upregulated in patients with lymph node metastasis and pathological stage IV (p < 0.01) (Fig. 2B). The CpG island methylator phenotype (CIMP) has been found to be strongly associated with poor prognosis in KIRP patients, and therefore SNHG6 expression levels in the CIMP group have been explored [19]. As expected, the expression of SNHG6 was significantly higher in the CIMP group than in classical type I and type II KIRP patients.

### The diagnostic and prognostic utility of SNHG6 in KIRP

**Fig. 3:**
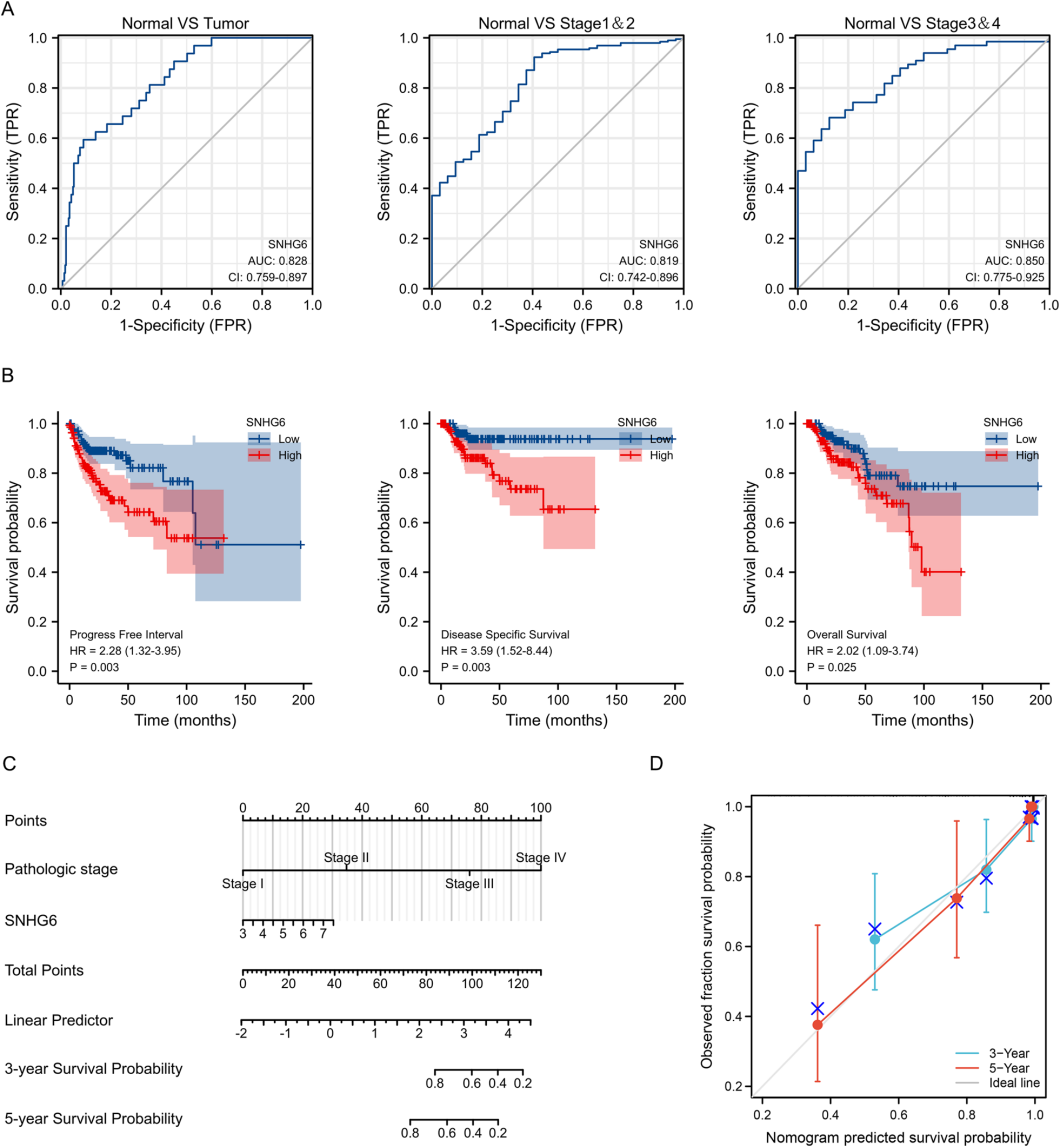
Diagnostic and prognostic value analysis of SNHG6. **A** ROC curves of SNHG6 expression in normal and tumor tissues. **B** Kaplan–Meier analysis showed that KIRP patients with high SNHG6 expression had worse OS, DSS and PFI. **C** Nomogram for predicting DSS at 3 and 5 years in KIRP patients. **D** Calibration curve to evaluate Nomogram consistency. SNHG6, Small nucleolar RNA host gene 6; KIRP, kidney renal papillary cell carcinoma; OS, overall survival; DSS, disease-specific survival; PFI, progression-free interval

ROC curves were used to assess the diagnostic efficacy of SNHG6 in KIRP. As shown in Fig. 3A, the AUC value for SNHG6 was 0.828 when differentiating KIRP tissue from normal tissue; for stage I-II and III-IV patients, the AUC values were 0.819 and 0.850, respectively. Kaplan–Meier curves visualised the impact of SNHG6 expression on oncological outcomes in KIRP patients; the high SNHG6 expression group was associated with worse PFI, DSS and OS in KIRP patients with hazard ratios (HR) of 2.28, 3.59 and 2.02 respectively (Fig. 3B) (p < 0.05). Subsequently, we implemented univariate and multivariate COX analyses to look for potential predictors of DSS, and pathological stage. SNHG6 expression were found to have non-negligible predictive value (Table 1). Accordingly, these two metrics were incorporated into the construction of the Nomogram to predict disease-specific survival at 3 and 5 years in KIRP patients (Fig. 3C). Encouragingly, the C-index was 0.920 (0.898–0.941) and the good agreement between predicted and actual values was also objectively demonstrated by the calibration curve in Fig. 3D.

**Table 1:** Univariate and Multivariate COX regression analysis for DSS

Characteristics	Total (N)	Univariate analysis		Multivariate analysis	
		Hazard ratio (95% CI)	P value	Hazard ratio (95% CI)	P value
Age	282				
< = 60	133	Reference			
> 60	149	0.442 (0.204–0.958)	**0.039**	0.493 (0.223–1.093)	0.082
Gender	284				
Female	77	Reference			
Male	207	0.558 (0.257–1.212)	0.140		
BMI	210				
< = 30	134	Reference			
> 30	76	0.597 (0.220–1.621)	0.311		
Race	268				
Asian&Black or African American	67	Reference			
White	201	0.922 (0.370–2.294)	0.861		
Smoker	243				
No	115	Reference			
Yes	128	0.602 (0.280–1.294)	0.194		
Pathologic stage	257				
Stage I&Stage II	191	Reference			
Stage III&Stage IV	66	42.300 (9.991–179.094)	** < 0.001**	41.137 (9.708–174.314)	** < 0.001**
SNHG6	284				
Low	143	Reference			
High	141	4.091 (1.658–10.094)	**0.002**	3.219 (1.287–8.055)	**0.012**

SNHG6, Small nucleolar RNA host gene 6; DSS, Disease-specific survival

### The relationship between SNHG6 and tumor-infiltrating immune cells

**Fig. 4:**
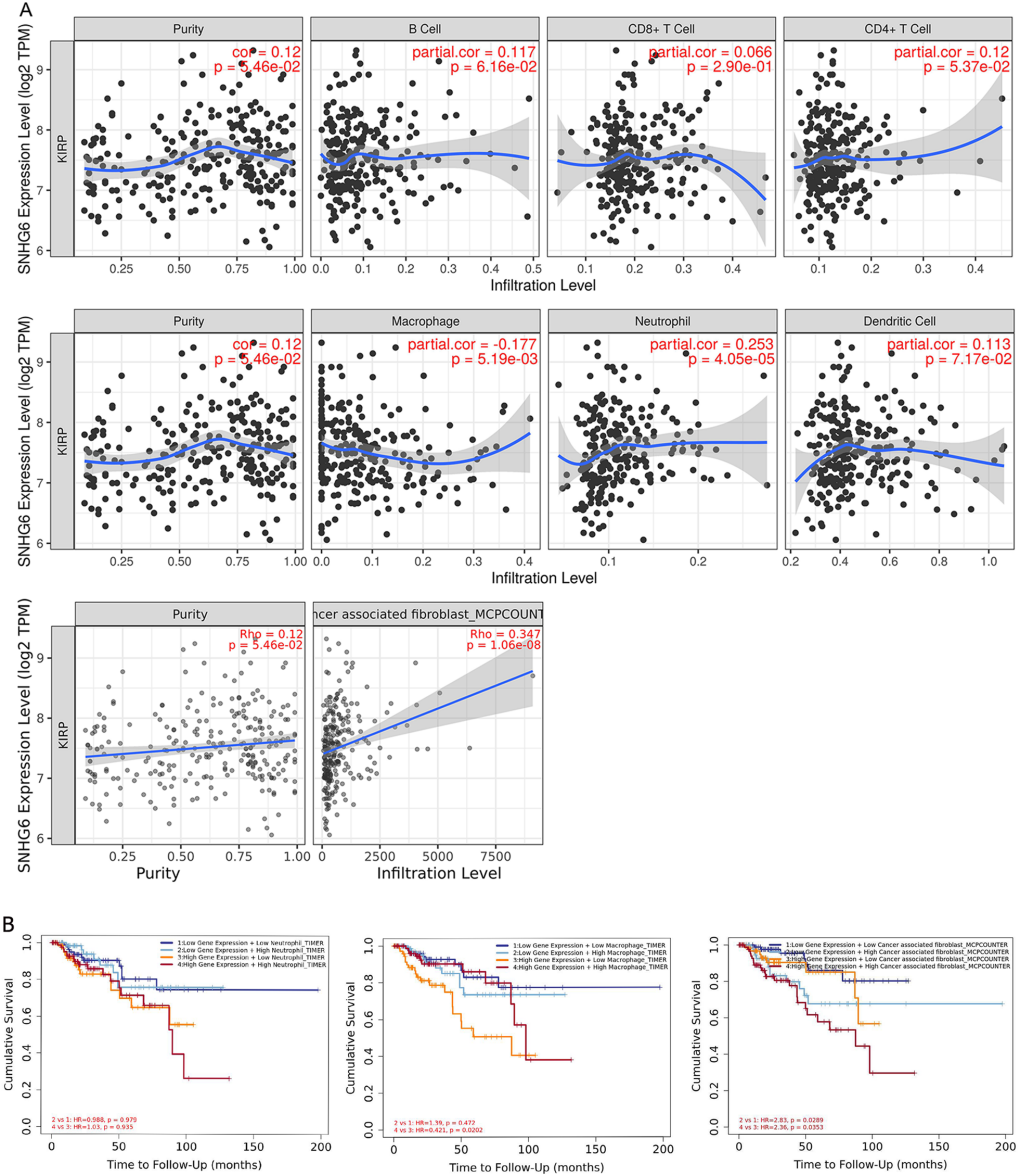
Analysis of the correlation between SNHG6 and tumor-infiltrating immune cells and the impact of this correlation on OS in KIRP patients. **A** There was no significant correlation between SNHG6 and B lymphocytes, T lymphocytes and dendritic cells, but a positive correlation with neutrophils and cancer associated fibroblasts, and a negative correlation with macrophages. **B** High cancer-associated fibroblast abundance and low macrophage abundance worsened OS in KIRP patients. SNHG6, Small nucleolar RNA host gene 6; KIRP, kidney renal papillary cell carcinoma; OS, overall survival

Using the TIMER database, we found that there was no significant correlation between SNHG6 and B lymphocytes, T lymphocytes and dendritic cells in the tumor microenvironment of KIRP, but was positively correlated with neutrophils and cancer associated fibroblasts, and negatively correlated with macrophages (Fig. 4A). To more visually demonstrate the significance of this correlation on the survival prognosis in KIRP patients, we combined those two variables to plot the Kaplan–Meier curves (Fig. 4B). Interestingly, lower macrophage abundance in the tumor microenvironment under high expression state of SNHG6 led to a deterioration of OS in KIRP patients. At the same time, we found that patients in the SNHG6 high expression plus high abundance of cancer-associated fibroblasts group had a worse OS than any other subgroup. As a result, the pro-oncogenic effect of SNHG6 on KIRP may be partially related to tumor-infiltrating immune cells, especially macrophages and cancer associated fibroblasts.

### mRNAs potentially regulated by SNHG6

**Fig. 5:**
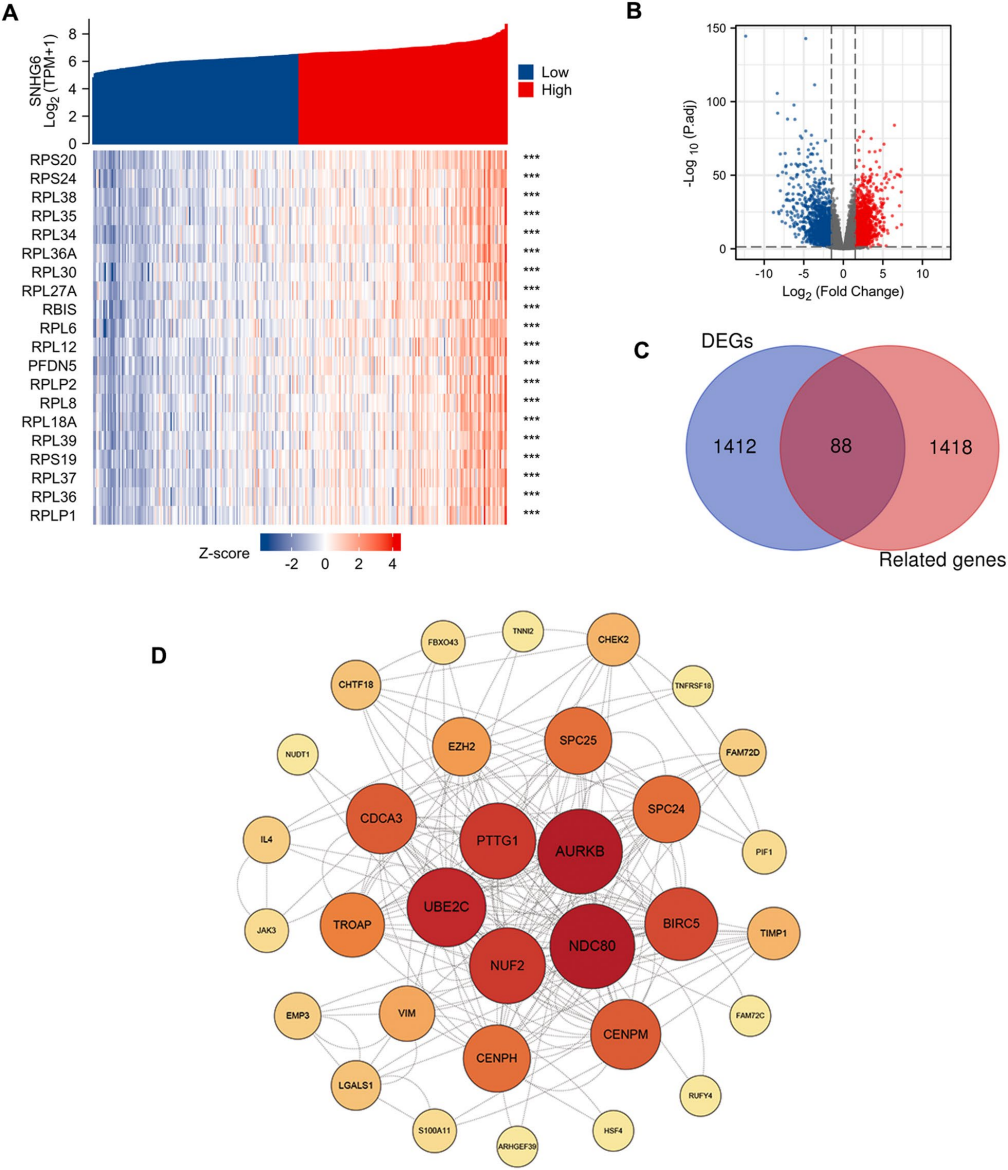
Screening of target mRNAs associated with SNHG6 **A** The top 20 mRNAs positively correlated with SNHG6. **B** A volcano map showing DEGs in the KIRP. **C** An intersection analysis of SNHG6-related genes and DEGs. **D** PPI network built with STRING database and Cytoscape. SNHG6, Small nucleolar RNA host gene 6; KIRP, kidney renal papillary cell carcinoma; PPI, Protein–protein interaction; DEGs: differentially expressed genes

**Fig. 6:**
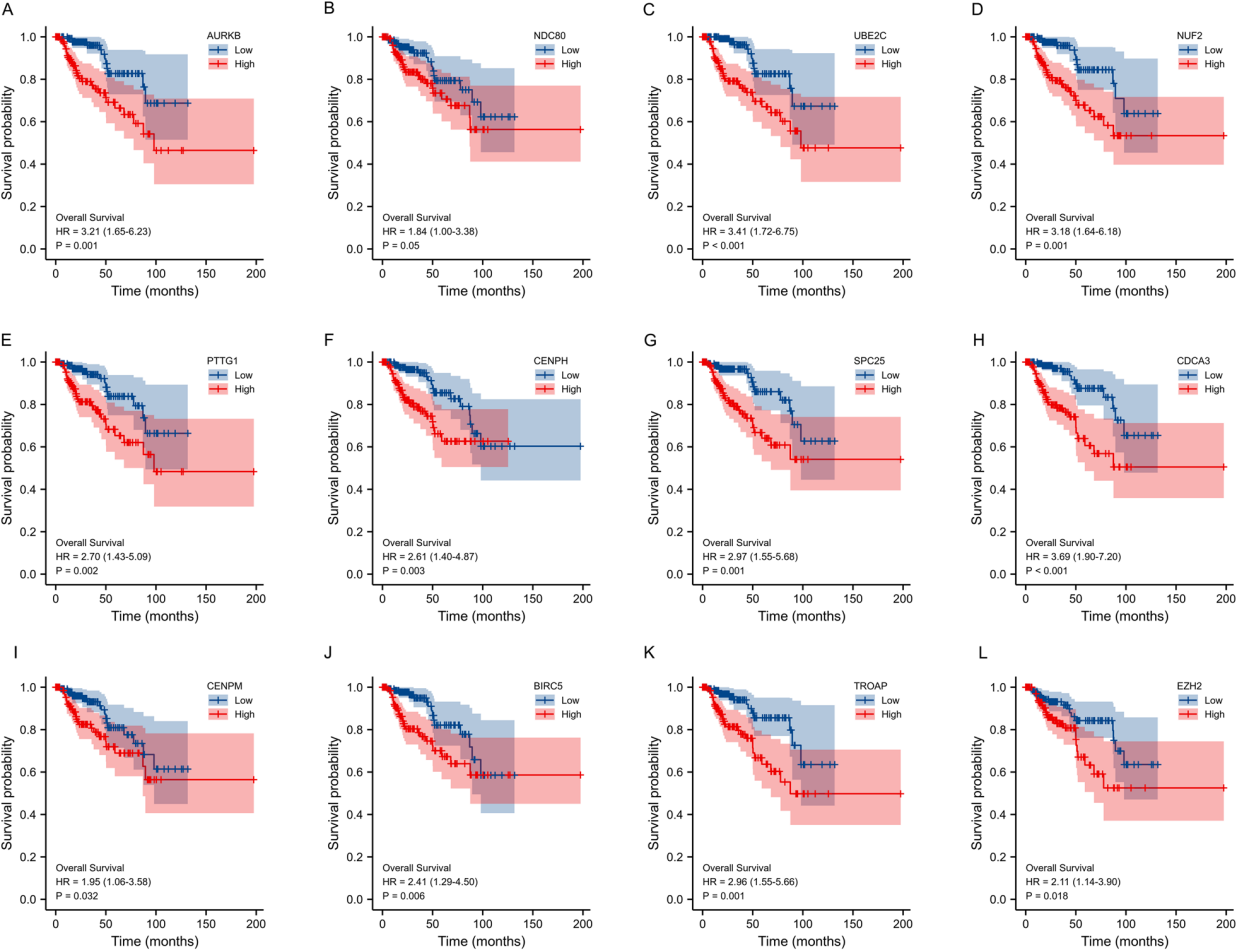
Kaplan–Meier analysis exploring the impact of hub gene expression on OS in KIRP patients. **A** AURKB. **B** NDC80. **C** UBE2C. **D** NUF2. **E** PTTG1. **F** CENPH. **G** SPC25. **H** CDCA3. **I** CENPM. **J** BIRC5. **K** TROAP. **L** EZH2. SNHG6, Small nucleolar RNA host gene 6; KIRP, kidney renal papillary cell carcinoma

Following the screening criteria, we found that SNHG6 was closely associated with 1506 mRNAs in KIRP, and the top 20 molecules have been shown via the heat map in Fig. 5A. On the other hand, differentially expressed mRNAs in KIRP were screened by the volcano map, where 1500 mRNAs were up-regulated (Fig. 5B). Venn diagram further identified overlapping genes in both groups (Fig. 5C). These 88 target molecules were then constructed into a PPI network through the STRING database and hub genes were screened based on the centrality of the nodes (Fig. 5D). Furthermore, Kaplan–Meier survival analysis was performed for mRNAs (AURKB, NDC80, UBE2C, NUF2, PTTG1, CENPH, SPC25, CDCA3, CENPM, BIRC5, TROAP, EZH2) with more edges to explore their impact on OS in KIRP patients. Intriguingly, the upregulation of almost all these molecules (except NDC80) in KIRP worsened the OS of the patients (Fig. 6A–L). Apparently, the adverse effect of these mRNAs on the survival prognosis of KIRP is in line with the trend of SNHG6.

### Exploration of the potential mechanisms of SNHG6 in KIRP

**Fig. 7:**
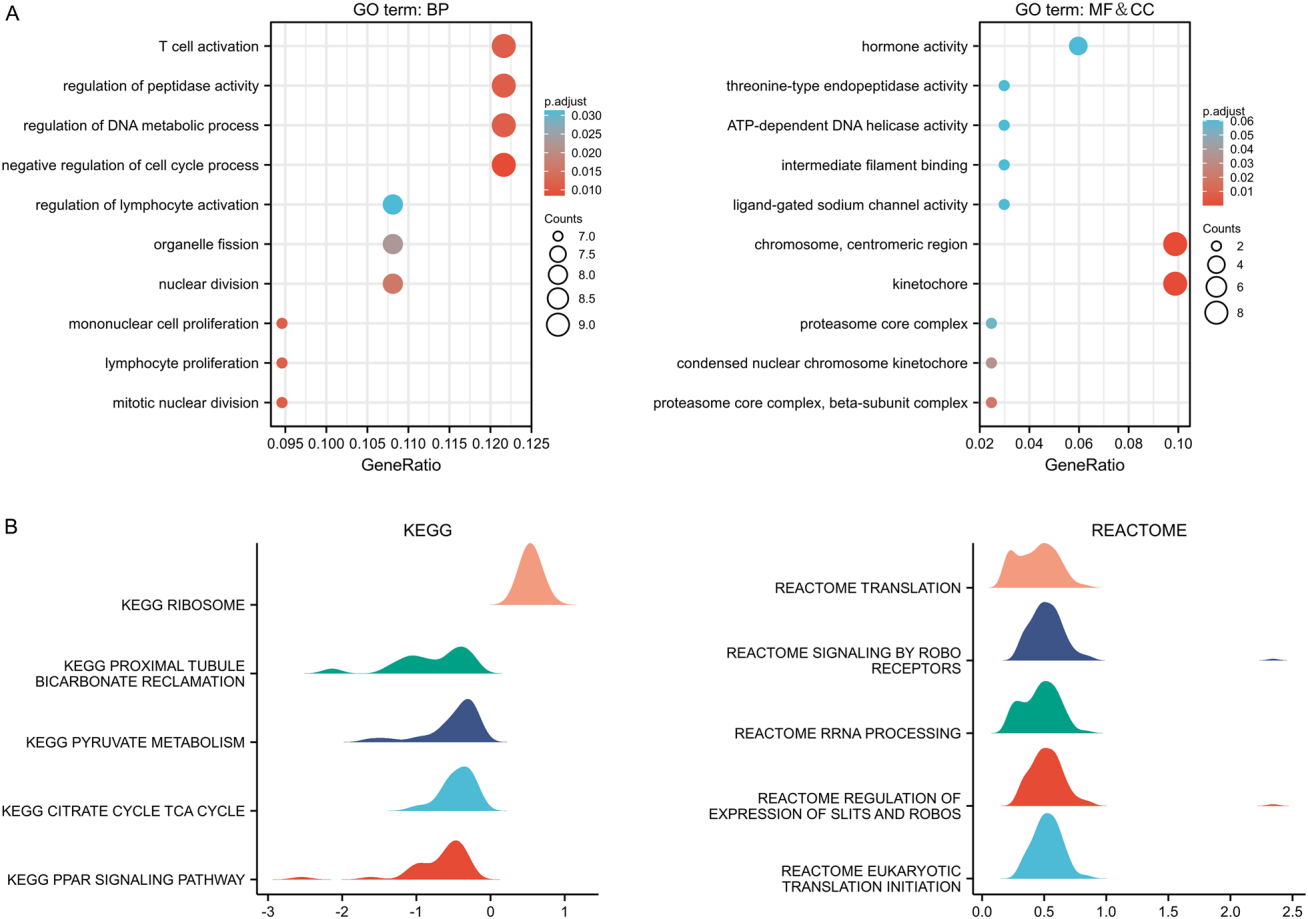
GO analysis and GSEA. **A** GO analysis: biological processes, molecular functions and cellular composition. **B** Enrichment plots by GSEA. GO, Gene Ontology. GSEA, gene set enrichment analysis

Based on the 88 target mRNAs obtained from the previous screening, we conducted GO analysis and found that SNHG6 may be involved in the following biological processes, including: T cell activation, regulation of peptidase activity, regulation of DNA metabolic process, negative regulation of cell cycle process, regulation of lymphocyte activation, etc. Meanwhile, it is involved in cellular components such as the chromosome centromeric region, kinetochore and proteasome core complex, and its potential molecular function is mainly to influence enzyme activity (Fig. 7A). In addition, GSEA was used to explore the potential signalling pathway of SNHG6 in KIRP. As shown in Figs. 7B, the KEGG pathways that were significantly enriched included the ribosome, proximal tubule bicarbonate reclamation, pyruvate metabolism, citrate cycle tca cycle, and peroxisome proliferator-activated receptor (PPAR) signaling pathway [20–22]. The REACTOME pathway includes translation, Roundabout (ROBO) signalling receptor transmission, RRNA processing, regulation of short-lived non-coding transcripts (SLITs) and ROBOs expression and initiation of eukaryotic translation.

## Discussion

KIRP is the second most common subtype of renal cell carcinomas and is characterized by the presence of basophilic or eosinophilic cells in papillary or tubular structures [23]. KIRP has long been considered a low-grade malignancy; and in patients with localized renal cell carcinomas, the papillary subtype is an independent prognostic factor for improved oncological outcome [24]. Conversely, a meta-analysis showed no significant improvement in the prognosis of patients with metastatic KIRP compared to patients with metastatic ccRCC, with patients with type II KIRP having an even worse outcome [25]. Furthermore, KIRP is a highly heterogeneous tumor and its prognosis is significantly associated with histological subtypes and genetic phenotypes. In this context, the treatment and management of advanced KIRP undoubtedly faces enormous challenges. Accordingly, more promising biomarkers or therapeutic targets need to be explored in the current work.

Human SNHG6 is the housekeeping gene of the 5′ TOP family, consisting of five transcripts, SNHG6-201 to SNHG6-205 [26]. High expression of SNHG6 has been found in a variety of cancer tissues (including colorectal cancer, hepatocellular carcinoma, breast cancer, ccRCC, etc.) and can predict a poor prognosis [11, 27–29]. In the present study, these results were also largely validated; in addition to this, we found that SNHG6 was also significantly upregulated in KIRP and strongly associated with unfavorable oncological outcomes. Patients with KIRP have a higher incidence of lymph node involvement compared to the metastatic pattern of ccRCC, which makes lymph node involvement more critical in the pathological grading system of KIRP [30]. Interestingly, our results showed that SNHG6 expression was positively correlated with lymph node metastatic lesions. Furthermore, we found that SNHG6 was significantly upregulated in KIRP patients with stage IV pathological stage and those carrying the CIMP phenotype. Not surprisingly, these results suggest that SNHG6 expression is associated with the more aggressive KIRP subtype.

To further explore the potential of SNHG6 as a biomarker for KIRP, we have comprehensively assessed its diagnostic and prognostic predictive value with the help of ROC curves and Kaplan–Meier analysis. As expected, SNHG6 showed good diagnostic performance and its up-regulated status predicted worse PFI, DSS and OS; this is in line with the findings of previous studies [9]. Subsequently, based on the results of the COX analysis, we constructed a Nomogram for predicting 3-year DSS and 5-year DSS in KIRP patients and showed satisfactory predictive efficacy. Consequently, based on the above data, there is reason to believe that SNHG6 is an independent predictor of poor prognosis in KIRP patients and may contribute in some way to the progression of KIRP.

Renal cell carcinoma has long been recognized as an immunogenic tumor and a variety of immune cells in the tumor microenvironment can be involved in mediating the anti-tumor immune response [31]. The presence of tumor-associated macrophages has been independently shown to reduce the risk of death in patients with KIRP [32]. Cancer associated fibroblasts have also been shown to play multiple roles in tumor development by secreting growth factors, inflammatory ligands and extracellular matrix proteins that promote cancer cell proliferation and immune rejection [33].According to the TIMER database, we found that SNHG6 expression in KIRP was negatively correlated with macrophage abundance and positively correlated with cancer associated fibroblasts. Furthermore, survival analyses further confirmed that this correlation have deleterious effects on OS in KIRP patients (Fig. 4B). Therefore, we speculated that SNHG6 may influence disease progression to some extent by participating in the regulation of the immune microenvironment of KIRP.

There is growing evidence that LncRNAs can be involved in pre-mRNA alternative splicing as transcriptional regulators in the nucleus on the one hand, and in post-transcriptional regulation (mRNA stability, mRNA translation, protein stability and ceRNA networks) in the cytoplasm on the other hand [34]. After qualifying the expression level of mRNAs with strong SNHG6 correlation in KIRP, we obtained 88 target molecules. Subsequently, we performed GO analysis using these 88 genes and the results showed that SNHG6 may be involved in a range of biological processes including: activation and proliferation of immune cells, regulation of peptidase activity and regulation of DNA metabolic process, etc. Encouragingly, these results seem to corroborate the previously mentioned role of SNHG6 in the regulation of the tumor immune microenvironment. Additionally, we performed PPI network construction using 88 target molecules and identified several hub genes such as AURKB, NDC80, UBE2C, NUF2, PTTG1, CENPH, SPC25, CDCA3, CENPM, BIRC5, TROAP, EZH2, etc. Also, upregulation of the expression of these molecules has been found to predict a poor prognosis for KIRP patients. Intriguingly, previous studies have shown that these molecules can contribute to the progression of a variety of cancers [35–42], with PTTG1 and CDCA3 being found to be prognostic biomarkers for KIRP [43, 44]. On the other hand, AURKB, BIRC5 and SPC25 were found to be significantly upregulated in CIMP-positive ccRCC tissues and were associated with poor prognosis [45]. EZH2 is considered to be a new target for cancer therapy and has been shown to be a downstream target gene regulated by SNHG6 in a variety of malignancies [11, 42, 46, 47]. Thus, given this favorable evidence, it seems reasonable to presume that SNHG6 may influence the biological behavior of KIRP by regulating the expression of these target genes.

Finally, we explored the potential mechanisms of SNHG6 in KIRP, and GSEA showed that SNHG6 is closely associated with the PPAR signalling pathway, Robo signalling receptor transmission and the regulation of Slit and Robo expression. To our knowledge, the PPAR signalling pathway can function pleiotropically in cancer, and PPARα antagonists can be involved in the regulation of multiple reprogrammed metabolic pathways and attenuate tumor growth in renal cell carcinomas [48, 49]. SLITs are a series of secreted proteins that regulate angiogenesis, inflammatory cell chemotaxis, tumor cell migration and metastasis by binding to ROBO receptors [50, 51]. However, GSEA can only provide researchers with preliminary evidence and specific signalling pathways will need to be further explored in subsequent studies.

It has to be mentioned that although this study systematically analyses the potential association of SNHG6 with the malignant phenotype of KIRP, it still has some shortcomings. For example, the data in this study were derived from public databases and lacked validation from the clinical sample in our study centre. Secondly, the exact mechanism of SNHG6 in the tumourigenesis development of KIRP remains unclear and needs to be refined in subsequent in vivo and in vitro experiments.

## Conclusion

Here, SNHG6 expression was found to be significantly upregulated in KIRP tissues and associated with a more aggressive KIRP subtype and poorer prognosis. Besides, SNHG6 may interfere with anti-tumor immune responses by affecting macrophages and cancer-associated fibroblasts in the tumor microenvironment, and may also promote KIRP progression by regulating the expression of molecules such as EZH2. Meanwhile, the PPAR signalling pathway and SLIT/ROBO signalling pathway may be specific signalling pathways for SNHG6 in KIRP.

## Data Availability

RNA-seq data for KIRP patients are available from both the TCGA database (https://portal.gdc.cancer.gov/) and the GTEx database (https://www.gtexportal.org/).
